# BRG1 and BRM loss selectively impacts RB and P53, respectively: BRG1 and BRM have differential functions *in vivo*

**DOI:** 10.18632/oncoscience.333

**Published:** 2016-12-21

**Authors:** Stefanie B. Marquez-Vilendrer, Sudhir K. Rai, Sarah JB Gramling, Li Lu, David N. Reisman

**Affiliations:** ^1^ Department of Hematology/Oncology, Medicine, University of Florida, Gainesville, FL, USA; ^2^ Department of Pathology, University of Florida, Gainesville, FL, USA

**Keywords:** retinoblastoma, lung cancer, p53, swi/snf, tumor suppressor

## Abstract

The SWI/SNF complex is an important regulator of gene expression that functions by interacting with a diverse array of cellular proteins. The catalytic subunits of SWI/SNF, BRG1 and BRM, are frequently lost alone or concomitantly in a range of different cancer types. This loss abrogates SWI/SNF complex function as well as the functions of proteins that are required for SWI/SNF function, such as RB1 and TP53. Yet while both proteins are known to be dependent on SWI/SNF, we found that BRG1, but not BRM, is functionally linked to RB1, such that loss of BRG1 can directly or indirectly inactivate the RB1 pathway. This newly discovered dependence of RB1 on BRG1 is important because it explains why BRG1 loss can blunt the growth-inhibitory effect of tyrosine kinase inhibitors (TKIs). We also observed that selection for *Trp53* mutations occurred in Brm-positive tumors but did not occur in Brm-negative tumors. Hence, these data indicate that, during cancer development, Trp53 is functionally dependent on Brm but not Brg1. Our findings show for the first time the key differences in Brm- and Brg1-specific SWI/SNF complexes and help explain why concomitant loss of Brg1 and Brm frequently occurs in cancer, as well as how their loss impacts cancer development.

## INTRODUCTION

The SWI/SNF complex is an essential regulator of a large cadre of genes. By moving and shifting the position of histones within the chromatin, SWI/SNF gives key cellular proteins and transcription factors access to specific DNA domains necessary to regulate gene expression [1]. This complex is composed of two mutually exclusive catalytic subunits, either Brahma (BRM or SMARCA2) or Brahma Related Gene 1 (BRG1 or SMARCA4), along with 8-10 subunits that assemble into at least three different but related SWI/SNF complexes [2, 3]. SWI/SNF subunits with similar functionality, such ARID1A versus ARID1B or BAF60A, B, and C versus BAF53 A and B, in combination with BRG1 or BRM, give SWI/SNF both the flexibility and diversity characteristic of the many different molecular complexes that can be assembled. Knowledge of the interplay of these subunits with cellular proteins is ever-expanding; however, it is not fully understood how the loss of SWI/SNF subunits truly impacts cancer development through the additional loss of expression of the other subunits, which are frequently altered in cancer.

Through its various protein interactions, the SWI/SNF complex has been linked to many cellular processes, including growth control, differentiation, development, adhesion, and DNA repair [4, 5]. It is not surprising, then, that this complex and its subunits are targeted during cancer development. Recent Next Generation (NextGen) sequencing studies show that at least one SWI/SNF subunit is mutated in 20% of all human cancers [6]. Certain subunits are highly mutated in specific cancers such as the following: *PBRM1* (*BAF180*) is mutated in liver and renal carcinoma [7, 8], while *ARID1A* is preferentially mutated in uterine, cervical, ovarian and gastric cancers [9-11]. The function of many of these subunits, however, is only partially understood in contrast to the function of *BRG1* and *BRM*. These subunits serve as the ATPase catalytic or mechanical motor of the SWI/SNF complex, and the loss of one or both clearly abrogates the ability of SWI/SNF to open the chromatin and change gene expression [4, 5]. This, together with the fact BRG1 and BRM are more broadly silenced in a range of cancers compared with other subunits, signifies that the study of the BRG1 and BRM subunits is important to the field of cancer research.

SWI/SNF, BRG1, and BRM have been linked to the function of a number of key cellular proteins required to thwart cancer development. In particular, SWI/SNF is known to be essential for RB1-mediated growth inhibition. Specifically, cell lines that lack both BRG1 and BRM are refractory to growth inhibition when a constitutively active form of RB1 is introduced [12, 13]. RB1-mediated growth inhibition can be restored if either BRG1 or BRM is also restored along with this introduction of RB1 [14, 15]. Moreover in BRG1/BRM-deficient cell lines, the re-expression of BRG1 or BRM readily stimulates growth inhibition [16-18], yet high levels of BRG1 or BRM used in *in vitro* experiments can activate both BRG1- dependent and BRM-dependent SWI/SNF complexes, and thus it is not clear whether RB1 is dependent on BRG1 or BRM complexes. This represents a major gap in our knowledge. Methods to restore BRG1 or BRM expression could be pursued as a novel avenue of targeted therapy, and therefore the determination of whether either BRG1- or BRM-dependent complexes or both are functionally tied to RB1 is critical. This is also potentially clinically important, as tyrosine kinase inhibitors (TKIs) arrest growth in part through the activation of RB1 [19], and knowledge of whether BRM, BRG1, or both is functionally tied to RB1 allows one to determine if the loss of either or both of these subunits might cause resistance to TKIs.

Similar to RB1, TP53 has been linked to the function of a number of subunits of SWI/SNF, including BRG1 and BRM [20, 21]. If BRG1 or BRM or both can substitute for *RB1* and *TP53*, one might predict an inverse relationship of the mutation rates of *TP53* and *RB1* with those of *BRG1* and *BRM*, as determined by NextGen sequencing studies. However, unlike *TP53*, which is principally inactivated by mutations, *BRM* is almost never mutated (<2%) but, rather, it is epigenetically silenced [22]. Similarly, the frequency of *BRG1* mutations is also relatively low (<2- 5%) in most tumors [22], and our recent work has shown that *BRG1* can be silenced by either aberrant splicing or translational blocking mechanisms regulated by the AKT pathway [23]. Interestingly, *ARID1A*, which is usually inactivated by mutation, does indeed show an inverse correlation with TP53 mutations in a number of tumor types [24-26]. Thus, like RB1, it is unclear if BRG1 or BRM-dependent complexes are functionally linked to TP53, or if both are linked to this protein. The oncogene- induced senescence hypothesis [27, 28] was designed to explain how cells develop into benign tumors and why they evolve into malignant tumors if and when both RB1 and TP53 proteins are abrogated. Since we know that SWI/SNF is functionally linked to both RB1 and TRP53, we predicted that tumors would more readily arise if BRG1 and BRM are concomitantly lost. Specifically, we wanted to determine if Brg1, Brm or both substitute for Rb1 and Trp53 in a murine model of lung cancer.

## METHODS

### Generation of murine lung adenocarcinoma

Quad mice (see tandem manuscript for definition of genotypes), which carried the CCSP-rtTA and (oTet) -Cre constructs (generous gifts from Jeff Whittsett [29, 30]) were used; these constructs drive the targeted inactivation of a floxed gene. Quad mice were homozygous for the *Brm*-null (*Brm*−/−) allele and for floxed-*Brg1* (*Brg1fLoxP*) alleles (gifts from Moshe Yaniv and Pierre Chambon, respectively) [31, 32]. We also generated Cre-negative mice, which were similar to Quad mice, but lacked the (oTet) -Cre construct and the *Brm*-null alleles. When the Quad mice were crossed with mice with Cre-negative phenotypes, an “Intermediate” strain resulted, in which mice were homozygous for the LoxP-*Brg1* gene and the CCSP-rtTA construct, but were hemizygous for the (oTet) -Cre constructs and the *Brm*-null allele. After these intermediate mice were crossed with each other and after the heterozygous *Brm*-null offspring were removed from further study, we generated four genotypes as follows: wild type (WT: *Brm+/Brg1+*), *Brm*-null (*Brm−/Brg1+*), *Brg1*-negative (*Brg1−/Brm+*), and double-negative (*Brg1−/Brm−*). At 6-7 weeks of age, all mice were given 2 intra- peritoneal (IP) injections (1 week apart) of 1 g/kg urethane (ethyl carbamate) to initiate tumor development. These injections also served to prevent apoptosis caused by Cre- mediated *Brg1* inactivation in normal lung cells (i.e., in type 2 alveolar and Clara cells) [33]. Four weeks after the first IP injection, the mice were provided *ad libitum* with water containing 1 mg/mL tetracycline and 3% sucrose for 5 days to induce Cre expression and thus inactivate the *Brg1* allele. At 6-12 months, the mice were euthanized due to the development physical stress, at which point lung adenocarcinomas were harvested and analyzed.

### Immunohistochemistry and Immunofluorescence

Hematoxylin & eosin (H&E) staining was performed to assess the general histology of the tumors and for scoring purposes. Antibodies to the following antigens were used in IHC/Immunofluorescence (IF) experiments: Brg1 (sc-374197, 1:50, Santa Cruz Biotechnology, Dallas, TX, USA); Brg1 (21634-1-AP, 1:250, Protein Tech, Chicago, IL, USA); Brm (1:200; rabbit polyclonal antibody generated by the Reisman Lab); Rb1 (ab6075, 1:100, Abcam, Cambridge, MA, USA); pRb1S780 (9307S, 1:200, Cell Signaling Technology, Danvers, MA, USA); pRb1T821/826 (sc-16669, 1:50, Santa Cruz Biotechnology, Dallas, TX, USA); Pcna (610664, 1:300, BD Biosciences, San Jose, CA, USA); Pcna (RB- 9055-P0, 1:200, Thermo Scientific, Waltham, MA, USA); Ki-67 (550609, 1:100, BD Biosciences); cyclin D1 (sc- 753, 1:50, Santa Cruz Biotechnology); Trp53 (sc-6243, 1:50, Santa Cruz Biotechnology); anti-Cdk2, GTX22363 (1:200, GeneTex); Cdk4 (12790, 1:100, Cell Signaling Technology). All antibodies were tested for cross- reactivity (specificity) by staining cell lines that lack the antigen of interest; these tests were then confirmed by western blot. All tissue sections were subjected to antigen retrieval, which consisted of heating in a microwave for 15 minutes on the high setting using either 10 mM sodium citrate buffer (pH 6), 10 mM Tris buffer (pH 10) or 10 mM Tris, 1 mM EDTA and 0.05% Tween20 (pH 8), depending on the antibody. Slides were incubated either overnight at 4°C or for 2 hours at room temperature. The appropriate biotinylated secondary antibodies were then used at a 1:200 dilution (BA-1000 or BA-9200, Vector Labs, Burlingame, CA, USA). This was followed by incubation with horseradish peroxidase streptavidin for 1 hour at room temperature (SA-5004, 1:200, Vector Labs). DAB was used as the chromogen (550880, BD Pharmingen, San Jose, CA, USA), and Harris hematoxylin was used as the counterstain. For IF experiments, the secondary antibodies Alexa-fluor 488 and Alexa-fluor 594 (A1100 and A21207, respectively, Life Technologies, Carlsbad, CA, USA) were used at dilutions of 1:200.

### Western Blot

Our western blot method has been described elsewhere [34, 35]. Briefly, the cells were harvested, and total protein was extracted using a urea-based lysis buffer, as described previously. Proteins were subjected to gel electrophoresis and then transferred onto polyvinylidene difluoride membranes. The membranes were incubated overnight at 4°C or at room temperature for 1 hour with the following primary antibodies: anti-Brg1, 21634-1- AP (1:1000, Protein Tech), phospho-Gsk3β (Ser9) (Cell Signaling Technology #9336), anti-Rb, sc-73598 (1:200, Santa Cruz Biotechnology), anti-p21, 2947P (1:1000, Cell Signaling Technology), anti-cyclin D1, 2978P (1:1000, Cell Signaling Technology), anti-Cdk2, GTX22363 (1:200, GeneTex) and anti-Gapdh, GTX100118 (1:1000, GeneTex). The pRb antibodies used in the western blot are the same as those described in the above section. The membranes were then incubated for 1 hour at room temperature with the appropriate biotinylated anti-mouse or anti-rabbit secondary antibody, which were used at a dilution of 1:2000 (GE Healthcare Life Sciences, Pittsburgh, PA, USA). Proteins were detected with a Chemiluminescence Western Bright ECL kit (K-12045-D50, Advansta Inc., Menlo Park, CA, USA).

### Sequencing

PFU enzyme and *Trp53* primers were used to amplify cDNA from the total RNA extracted from the tumors. Similarly, individual cDNA clones were amplified with *Pfu* polymerase, A-tailed and sequenced using M13 forward and reverse primers following cloning into a pGEM T Easy vector. (Note: *Pfu* produces blunt ends so that the PCR product must be A-tailed for cloning into T vector). Bands were isolated, gel-purified (QIAQuick Gel Extraction Kit, Qiagen, Valencia, CA, USA) and submitted for Sanger sequencing at the sequencing core of the Interdisciplinary Center for Biotechnology Research at the University of Florida.

## RESULTS

### *Trp53* mutations are not detected in tumors derived from *Brm*-null mice

While activated oncogenes drive the initial development of lung adenomas, senescence of these premalignant growths is known to be induced by Rb1 and Trp53, which block their further transformation into adenocarcinomas [36]. As such, the transformation from adenoma to adenocarcinoma occurs via the abrogation of the senescence effects of Trp53 and Rb1 [27, 28, 37]. However, if Brg1 and Brm are indeed necessary cofactors for the function of Rb1 and Trp53, we would expect that this transformation would also be fostered by the inactivation *Brg1*, *Brm* or both. Previous studies have established a dependency of the apoptotic and cell cycle arrest functions of Trp53 upon interaction with and specific binding to SWI/SNF subunits [20, 38-40]. However, it is unclear if Trp53 dependence on SWI/SNF stems from its interaction with Brg1- or with Brm-dependent complexes. As mutations in *Trp53* are commonly found in murine lung adenocarcinomas, we investigated whether there was a change in the observed *Trp53* mutation rate in these lung adenocarcinomas as a function of Brm or Brg1 loss.

**Figure 1:**
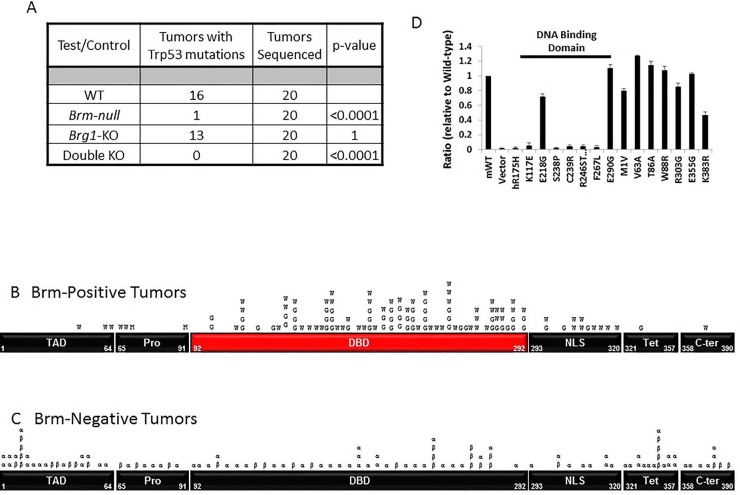
Figure 1A shows the results from sequencing 20 adenocarcinomas from each of the four different mouse phenotypes The tumors from Brm-positive mice (wild type and *Brg1* knockdown phenotypes) harbored 16/20 and 13/20 *Trp53* mutations, respectively, while tumors from Brm-negative mice (*Brm*-null or double knockout phenotypes) harbored 1/20 and 0/20 *Trp53* mutations, respectively. Figures 1B and 1C show the distribution of *Trp53* mutations along the *Trp53* cDNA from Brm-positive tumors and Brm-negative tumors. The majority of mutations in the Brm-positive tumors are distributed within the Trp53 “hot spot” or DNA binding domain (DBD), while the *p53* mutations from the Brm-negative tumors are distributed along the *Trp53* cDNA: W=wild type, G=*Brg1*-KO, α=*Brm*-null and β=double knockout. Figure 1D fourteen different *Trp53* cDNA clones found in both tumors from wild type and *Brm*-null phenotypes were introduced into the TRP53-negative cell line along with a luciferase TP53 reporter construct. The *Trp53* mutations from the DNA binding domain lacked Trp53 transcription activity as measured by this TP53 luciferase reporter as compared to those *Trp53* mutations derived from the other Trp53 domains.

In our murine lung cancer model, we observed malignant adenocarcinomas in all four genotypes used in this experimental system, with a frequency of 99%, 80%, 73% and 48% for the DKO, *Brg1*-KO, *Brm*-null and wild type genotypes, respectively (as described in our tandem paper). To determine possible functional relationships between Brg1 and Brm proteins with Trp53, we determined the rate of *Trp53* mutations in each of the four genotypes by sequencing the total mRNA from these tumors. The sequencing of *Trp53* (n=20 for each genotype) revealed that 80.0% (16/20) and 66.6% (13/20) of tumors from WT and *Brg1*-KO genotypes (Brm- positive tumors), respectively, harbored *Trp53* missense mutations (Figure 1A); differences in the mutation rates between these two groups were not statistically significant (p>0.05). In comparison, *Trp53* mutations occurred in only 5% (1/20) and 0% (0/20) of tumors from *Brm*-null and DKO mice (Brm-negative tumors), respectively (Figure 1A). The difference between the *Trp53* mutation rates in tumors from the Brm-positive and Brm-negative phenotypes was statistically significant (p<0.01). Of the mutations found in the WT (∼69) and *Brg1*-KO (∼63) tumor cells, we determined that 56/69 (∼80%) and 47/63 (∼75%) *Trp53* mutations occurred between amino acids 100-300, which is the DNA binding domain (or the *TRP53* mutation hot spot in humans). The observed lack of *Trp53* mutations in the *Brm*-negative phenotype was not likely due to loss of Trp53 expression via *p19Arf1* mutations or *Mdm2* amplifications, since Trp53 was expressed in the vast of majority of tumor cells (>85%) by IHC and was not qualitatively different between Brm-negative tumors and Brm-positive tumors (Supplementary Figure 1). Interestingly, the lack of observable *Trp53* mutations has also been shown in ovarian and gastric tumors that also demonstrate the loss of another SWI/SNF subunit, ARID1A [24-26].

#### Brm loss blocks selection of *Trp53* mutations

The above data do not preclude the possibility that *Trp53* mutations develop within tumor cells, but rather, the data suggest that such mutations might occur in less than 5% of tumor cells and thus are not detectible via Sanger sequencing. To test this hypothesis, we sequenced individual *Trp53* cDNA clones (70 each) from WT- and *Brm*-null-derived tumors. We observed similar frequencies of non-synonymous (missense) *Trp53* mutations of 79% and 58% from the WT and *Brm*-null phenotypes, respectively (p>0.05). These mutations that were detected in individual cDNA clones from tumors derived from the *Brm*-null phenotypes appeared to be randomly distributed throughout the *Trp53* open reading frame (Figure 1C). In contrast, the *Trp53* mutations that were detected by Sanger sequencing (cDNA clones and total mRNA) from wild type *Brm*-positive tumors appeared to be clustered in a known *Tp53* mutational hotspot DNA binding domain (DBD) (Figure 1B). These data suggest that *Trp53* mutations arose in individual tumor cells in mice of both Brm-positive and Brm-negative phenotypes, but that in the Brm-positive tumor cells, the clones of more dysfunctional *Trp53* mutations became more ubiquitous (positive selection) within a given Brm-positive tumor. As the percentage of cells with these mutations increased within the tumors (>5-10% of the tumor), this in turn allowed the mutations to become detectible by standard Sanger sequencing.

We sought to determine whether *Trp53* mutations within the DBD in general lacked function regardless of whether they were derived from either *Brm* genotype; similarly, we sought to determine whether *Trp53* mutations from the other domains retained TRP53 function. We tested 14 clones identified from both Brm- negative and Brm-positive tumors. Of these clones, we tested 7 from the DNA binding domain and 7 from outside this region (Supplementary Figure 2). As expected, the *Trp53* mutant cDNA clones from the DBD showed little to no transcriptional function in a Trp53-dependent luciferase reporter assay, regardless of the genotype of origin. In comparison, the cDNA clones from outside the DBD largely retained Trp53 function (Figure 1D and Supplementary Figure 2). Therefore, the more tumorigenic mutations from the DBD only arose by selection if they occurred in Brm-positive tumors. In contrast, *Trp53* mutations that arose from all regions in Brm-negative tumors, regardless if they caused an inactivation of Trp53 function, failed to undergo selection and did not result in an increase in the percentage of tumor cells expressing a given *Trp53* mutation. Based on these data, loss of Brm expression causes loss of *Trp53* selection/evolution during tumor development.

### Loss of Brg1 substitutes for Rb1 inactivation, but Brm loss does not affect Rb1 phosphorylation

We next examined whether loss of Brg1, Brm, or both affected how the Rb1 pathway became inactivated. *In vitro* data have shown that BRG1 and BRM are necessary cofactors for RB1 function, where RB1 poorly inhibits growth in the absence of a functional SWI/SNF complex, and where the re-expression of either BRG1 or BRM in BRG1/BRM-deficient cell lines restores RB1- mediated growth inhibition [14, 15]. Although these *in vitro* experiments demonstrate the functional dependency of RB1 on either BRG1 or BRM, it is not clear if RB1 specifically depends on BRG1- or BRM-specific complexes *in vivo*.

Using these lung tumor phenotypes, we sought to determine whether Rb1 is functionally linked to Brg1, Brm or both by examining the Rb1 phosphorylation status as a function of Brg1/Brm expression. To accomplish this, we performed dual immunofluorescence (IF) staining using an anti-Brg1 or anti-Brm antibody with two different anti-phosphoRb1 (pRb1) antibodies to reflect Rb1 activity or inactivity. Specifically, hyperphosphorylation at Rb1- Ser780 (pRb1S780) occurs by mitogenic stimulation, while hypophosphorylation (pRb1hypo) occurs during G1 arrest [41]. Phosphorylation at Rb1-Thr821/826 (pRB1T821/826) causes a conformational change, which prevents the binding of Rb1 to proteins that contain the LXCXE sequence [42]. Therefore, these two phospho-Rb1 antibodies give an indirect measurement of Rb1 functionality or lack thereof.

#### Brm loss does not correlate with Rb1 phosphorylation

**Figure 2:**
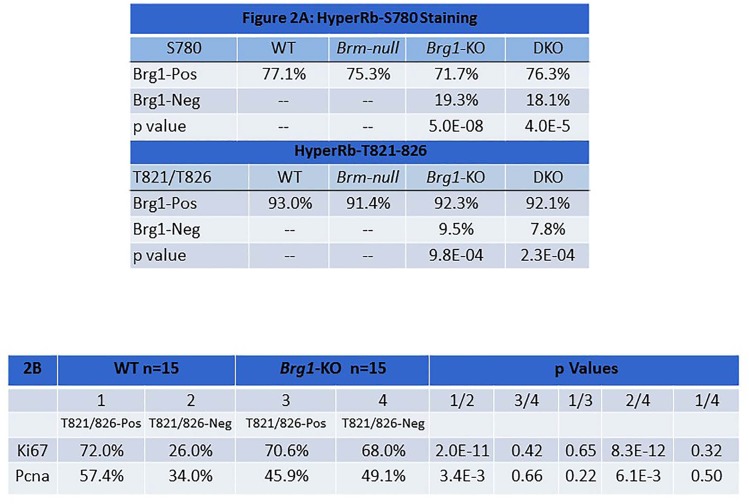
Figure 2A shows the results of dual IF staining with anti-Brg1 and anti-pRb1^S780^ (top) or anti-pRb1^T821/826^ (bottom) in lung tumors derived from each of the four genotypes The results from Brg1-positive tumor cells (top row) are compared with results from Brg1-negative tumor cells, and the p values (bottom rows) from the comparisons are given. Figure 2B shows the results for dual IF of pRb1T821/826 with anti-Ki67 (top row) and with Pcna (bottom row). P values that compare the percentage of positive staining of Ki67 and Pcna when pRb1T821/826 staining is either positive or negative are given.

We first conducted IHC for total Rb1 expression to exclude the loss of Rb1, and we observed that Rb1- negative tumor cells accounted for less than 5% of all tumor cells in tumors from each of the four genotypes, which is consistent with previously published reports [43]. Next, dual IF staining was conducted with anti-Brm and anti-pRb1S780 antibodies to determine the phosphorylation state of Rb1 as a function of Brm expression. We found that the frequencies of Rb1 hyperphosphorylation in Brm- positive (WT mice) and Brm-negative tumors (*Brm*-null mice) were similar (∼75%; p>0.05) (Figure 2A, top). Similarly, using antibodies to Brm and pRb1T821/826, we found no significant difference in pRb1T821/826 staining (∼90% p>0.5) as a function of Brm expression (Figure 2A, bottom). Hence, Brm loss did not affect or change the phosphorylation status of Rb1.

#### Brg1 loss is linked to the hypophosphorylation of Rb1

We then examined the phosphorylation status of Rb1 in Brg1-positive tumor cells using dual IF with antibodies against Brg1 and pRb1*S780* in tumors from each of the four genotypes. Although *Brg1*-KO tumors were not necessarily completely devoid of Brg1 expression, the majority of tumor cells from these genotypes were Brg1-negative (variable mosaic pattern). By dual IF, we observed the frequency of pRb1S780 staining to be significantly and statistically lower in Brg1-negative tumor cells derived from *Brg1*-KO mice compared with Brg1- positive cells from WT tumors (19.3% vs. 77.1%: p=5E-8) (Figure 2A). In fact, Brg1-positive cells in tumors from each of the four genotypes showed significantly more pRb1S780 immunoreactivity than Brg1-negative tumor cells from *Brg1*-KO mice (p<0.0001). In a parallel experiment, dual IF with antibodies against Brg1 and pRb1T821/826 was conducted. With this pRb1 antibody, we again obtained statistically significant results showing that Rb1 was more likely to be hypophosphorylated (i.e., in its active state) in Brg1-negative cells (*Brg1*-KO) compared with Brg1-positive (WT) cells (Figure 2A) (9.5% vs. 92.3%: p=9.8E-4). Importantly, IHC confirmed the infrequent loss of total Rb1 in these tumors (<5% of tumor cells in a given tumor were negative for Rb1) (data not shown). As Rb1 is not frequently lost in these tumors, these data demonstrate that Brg1 loss correlates with the hypophosphorylated state of Rb1.

#### Is Brg1 loss sufficient to disrupt the Rb1 pathway?

The loss of p16 (CDKN2A) and the overexpression of cyclin D/E and/or Cdk2/4 drive inactivation of the Rb1 pathway by causing Rb1 to become hyperphosphorylated (pRB1Hyper). Brg1-negative tumor cells primarily harbored hypophosphorylated Rb1 (pRb1Hypo), so none of the above mechanisms of Rb1 inactivation (pRb1Hyper) accounts for how the Rb1 pathway is inactivated in Brg1-negative tumor cells. Additionally, the infrequent loss of total Rb1 in these tumors eliminates that as a major cause for disruption of the Rb1 pathway. Previous work by multiple investigators has shown that Rb1-mediated growth inhibition requires a functional Brg1 protein [12-15]. As such, the *in vivo* loss of Brg1 might be sufficient to inactivate the Rb1 pathway. As shown in Figure 2A, we observed that only ∼19% and ∼9% of Brg1-negative tumor cells were also positive for pRb1S780 and/or pRb1T821/826, respectively, by dual IF; in other words, ∼80% and ∼90% of Brg1-negative cells failed to express either pRb1S780 or pRb1T821/826 and also harbor hypophosphorylated or activated Rb1, which should inhibit growth. We hypothesized that if Brg1 loss does indeed inactivate the Rb1 pathway, Brg1-negative cells should continue to proliferate even though they harbor active Rb1. To test this hypothesis, we needed to determine definitively whether these pRb1Hypo-positive/Brg1-negative tumor cells were proliferating and were not growth-arrested.

#### Is Pcna/Ki67 expression similar in pRb1^Hypo^-expressing Brg1-KO tumor cells and in pRb1^Hyper^-expressing WT tumor cells?

In order to determine if Brg1-negative/pRb1Hypo tumor cells were proliferating or if they were growth- arrested, we conducted dual IF to detect two different markers of proliferation (Ki67 or Pcna) together with the anti-pRb1T821/826 antibody. We compared the percentage of Pcna immunoreactivity in WT tumor cells that expressed pRb1Hyper (inactive Rb1) and those that did not (i.e., expressed pRb1hypo) (Figure 2B). We observed that Pcna staining was significantly higher at 57.4% in WT pRb1Hyper-Brg1 positive tumor cells versus 34% (p=3.4E-3) in pRb1Hypo-Brg1-positive WT tumor cells. This indicates that in WT tumor cells, when Rb1 is hypophosphorylated, tumor cells grow more slowly. In comparison, in Brg1-KO tumor cells, we found no difference in Pcna immunoreactivity between pRb1T821/826-positive and pRb1T821/826-negative tumor cells (45.9% vs. 49.1%, respectively, p=0.66). We observed a similar pattern with antibodies against Ki67 and anti-pRb1T821/826 (Figure 2B). A statistically significant decrease in Ki67 immunoreactivity was observed in the WT Brg1-positive tumor cells that did not express pRb1T821/826 compared with WT Brg1- positive tumor cells that did express pRb1T821/826 (positive) (26% and 72.0%, respectively; p=2.0E-11). Again, this finding indicates that WT tumor cells that express pRbHypo grow more slowly when Brg1 is present. In comparison, in the *Brg1*-KO tumor cells, we did not observe a statistically significant difference in Ki67 staining between pRb1*T821/826*-negative and pRb1*T821/826*-positive tumor cells (70.6% vs. 68.0%, respectively, p=0.42). Our results indicate that proliferation of WT Brg1-positive tumor cells is slowed in the presence of pRb1*Hypo* while Brg1-deficient tumors grow equally well in the presence of either pbB1*Hypo* or pRb1*Hyper*.

#### BRG1 loss regulates the phosphorylation of RB1

**Figure 3:**
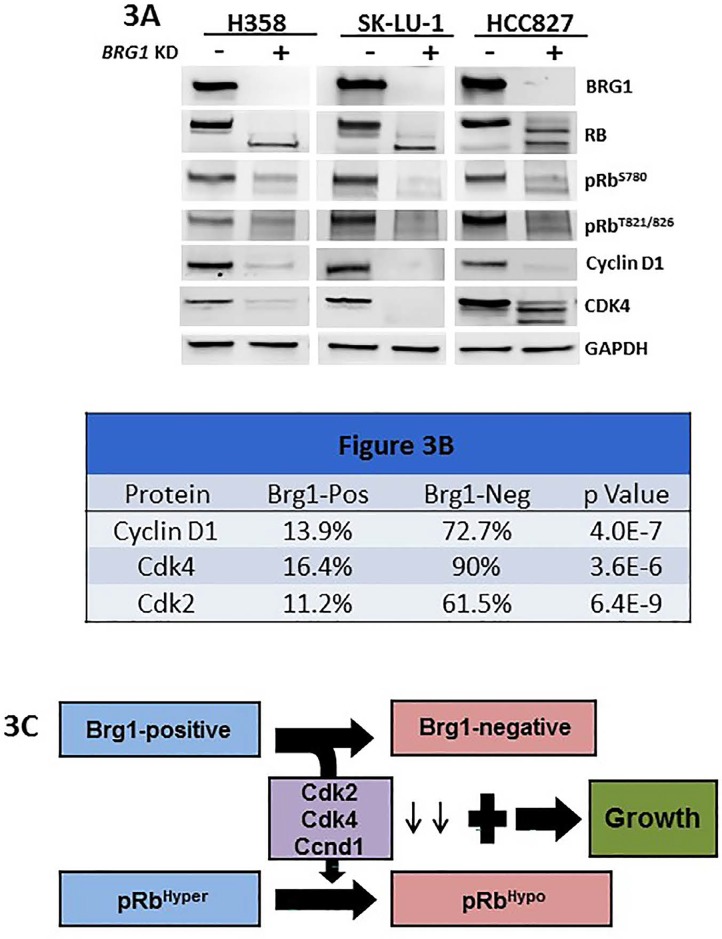
Figure 3A shows the protein expression levels as measured by western blot with antibodies to BRG1, RB1, pRB1^S780^, pRB1^T821/826^, CCND1, CDK4 and GAPDH for the three human cell lines H358, SK-LU-1 and HCC827 with (+) and without (−) *BRG1* knockdown Also shown is the effect of *BRG1* suppression by shRNA knock down on RB1 phosphorylation. Cell proliferation persists (before or after *BRG1* knock down) since either RB1 or BRG1 is inactivated and therefore cannot cooperate to foster RB1-mediated growth inhibition in either situation. Figure 3B shows the results of dual IF for Brg1 and Ccnd1 in the top row and the results for dual IF between Brg1 and Cdk4 and Brg1 and Cdk2 in the middle and bottom rows, respectively; p values for these comparisons are given in the column on the far right. Figure 3C illustrates the changes in Cdk2, Cdk4 and Ccnd1 as a function of Brg1 loss and how the loss of Brg1 potentially allows the Brg1-negative tumor cells to continue to grow, despite being in its active growth-inhibitory state.

The connection between BRG1 loss and RB1 phosphorylation suggests a relationship between these two proteins. Hence, we examined the impact of BRG1 knockdown via an shRNA approach in 3 lung cancer- derived cell lines (H358, SK-LU-1 and HCC-827) to determine the potential effects on RB1 phosphorylation. By western blot, BRG1 knockdown caused an observed shift from upper phosphorylated bands to lower non- phosphorylated bands, which indicates a change from an inactive RB1 (pRB1*Hyper*) state to an active one (pRB1*Hypo*), as well as a decrease in phosphorylation at RB1 sites T821/826 and S780. BRG1 knockdown also caused the downregulation of CCND1 (cyclin D1), CDK2 and CDK4 (Figure 3A), which has been previously reported [44-47]. The expression of CCNE1, CDKN1A (p21) and CDKN2A (p16) either did not appear to change or did not change in a similar direction in all 3 lung cancer cell lines as a function of BRG1 expression (data not shown).

Next, we analyzed the expression of these cell cycle- associated genes by qPCR in tumors derived from WT and *Brg1*-KO mice. While we observed little to no difference in the expression levels of *p16* (*Cdkn2a*) and *Ccne1* in tumors of these genotypes, we did observe a decrease in *Cdk4* (4.8-fold p<0.01) and a decrease in *Ccnd1* mRNA (∼5.8-fold; p<0.01) in the *Brg1*-KO tumor cells compared with the WT tumor cells. To confirm that changes in *Ccnd1* and *Cdk4* contribute to the decrease in Rb1 phosphorylation in murine Brg1-deficient lung tumors, we conducted dual IF for Brg1 versus both Ccnd1 and Cdk4. We observed that Ccnd1 was expressed in 13.9% of Brg1-negative tumor cells and was expressed in 72.7% of Brg1-positive tumor cells; this difference was statistically significant (p=4.0E-7) (Figure 3B). Similarly, Cdk4 was expressed in 16.4% of Brg1-negative tumor cells and in 90% of Brg1-positive tumor cells; this difference was also statistically significant (p=3.6E-6) (Figure 3B). In addition, by dual IF, we observed a statistical correlation between Cdk2 loss and Brg1 loss (11.2% and 61.5% in *Brg1*-KO and WT tumors, respectively; p=6.4E-9). These data indicate that Brg1 loss is linked to pRb1Hypo in part through decreases in Cdk2, Cdk4 and Ccnd1 expression (Figure 3C). In addition, the observed decrease in Rb1 phosphorylation in these murine adenocarcinoma cells was not associated with a decrease in the expression of the proliferation markers Pcna and Ki67, which indicates that the Rb1 pathway is likely unable to inhibit growth. These data therefore suggest that Brg1 loss indirectly or directly blocks the function of the Rb1 pathway, which allows cancer cells that express pRb1Hypo to continue to proliferate. This is consistent with *in vitro* data that show that Brg1 is a required cofactor for Rb1-mediated growth inhibition in human tumor-derived cell lines.

### Brg1 loss blocks tyrosine kinase inhibitor-induced RB1-mediated growth inhibition

**Figure 4:**
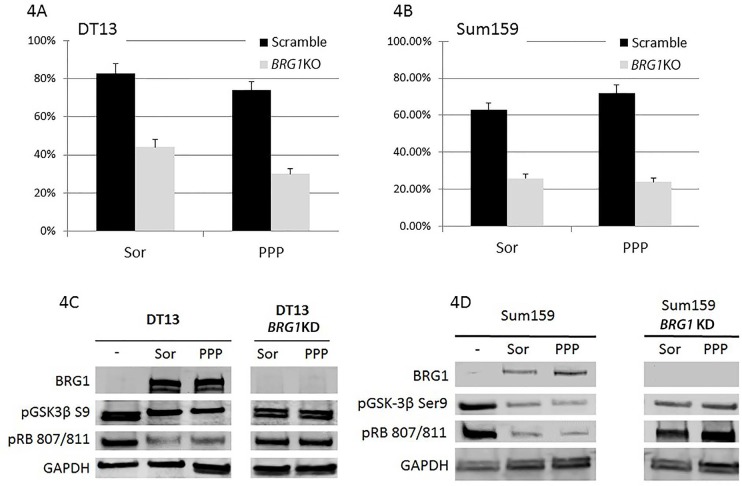
Figure 4A and 4B: DT13, a BRG1-deficient breast cancer cell line, was stably transduced with scrambled shRNA or anti-*BRG1* shRNA Each of the resultant four daughter cell lines were treated with 5 μM Sorafenib or 2 μM Picropodophyllin over 96 hours. The degree of growth inhibition after Sorafenib or Picropodophyllin (PPP) treatment was measured and is shown in Figure 3A (DT13) and Figure 3B (Sum159) for each of these cell lines expressing either scrambled or anti-*BRG1* shRNA. Figure 4C shows that the DT13 cell line was stably transduced with either scrambled or anti-*BRG1* shRNA. Each of these two daughter cell lines was then treated with Sorafenib or PPP, and after 96 hours, protein was extracted and a western blot was performed using antibodies against BRG1, GSK3β and GAPDH (loading control). The first lane indicates the vehicle-only- treated (DMSO) DT13 cells harboring the scrambled shRNA. Lanes 2 and 3 contain DT13 cells harboring scrambled shRNA and treated with Sorafenib or PPP, respectively. Lanes 4 and 5 contain DT13 cells treated with Sorafenib or Picropodophyllin, respectively; however, these cells also harbored anti-*BRG1* shRNA, which blocked the induction of BRG1. GSK3β decreases with the treatment of these TKIs. However, BRG1 knockdown appears to block the TKI-driven downregulation of pGSK3β expression. Figure 4D shows Sum159, another BRG1-deficient cell line, that is stably transduced with either scrambled or anti-*BRG1* shRNA. Lane 1 shows the Sum159 cells treated with vehicle-only scrambled shRNA. Lanes 2 and 3 show the two daughter Sum159 cell lines treated with scrambled shRNA and with Sorafenib or Picropodophyllin, respectively. Similarly, lanes 4 and 5 shows the Sum159 cells transduced with anti-*BRG1* shRNA and after treatment with Sorafenib or Picropodophyllin. Sor= sorafenib and PPP= Picropodophyllin. GAPDH was used as the loading control.

To date, the RB1 pathway in human cancers has been reported to be disrupted only by loss of expression of CDKN2A or RB1, as well as by CDK4/CCND1 over- expression [48], even though SWI/SNF is known to be an essential cofactor for RB1 function [16, 17]. This is due in part because it has never been clear whether BRG1 or BRM is essential for RB1, although both have been shown to be functionally linked to RB1 [14, 15]. Based on the understanding that RB1 is essentially only connected with BRG1, we could now pursue the impact of BRG1 loss on RB1-dependent processes. To this end, most TKIs inhibit growth in part by activating RB1- mediated growth inhibition [49, 50]. One would therefore predict that loss of SWI/SNF complex activity, and more specifically the loss of BRG1 expression, would abrogate the ability of RB1 to inhibit growth and thereby block the general ability of TKIs to halt tumor growth. To test this prediction, we tested two TKIs, Sorafenib (a RAF- 1, B-RAF and VEGFR-2 inhibitor) and Picropodophyllin (PPP, an inhibitor of insulin-like growth factor receptor: IGF-1R). We treated two BRG1-deficient cancer cell lines that harbored either scrambled or anti-*BRG1* shRNA with either Sorafenib or PPP for 96 hours and measured the expression of BRG1 and the number of cells over time to determine the growth rate. We observed that both compounds readily induced BRG1 as well as reduced GSK3β phosphorylation (Figure 4C and 4D), and inhibited the growth of Sum159 and DT13, two BRG1- deficient breast cancer cell lines (Figure 4A and 4B). However, if these cell lines harbored anti-*BRG1* shRNA, we observed that BRG1 was not induced and GSK3β phosphorylation was virtually unchanged, as measured by western blot (Figure 4C and 4D). This lack of change in GSK3β phosphorylation is important because the hypo- phosphorylated GSK3β is the active form, which fosters CCND1 degradation and causes p21 to translocate to the nucleus [51, 52]. Both of these changes cause RB1 to become hypophosphorylated and active, thereby inhibiting growth. Hence, the lack of BRG1 expression blocks RB1- mediated growth inhibition by two different possible mechanisms. First, the lack of BRG1 could directly or indirectly block GSK3β dephosphorylation, which in turns inhibits the dephosphorylation and activation of RB1. This can be seen in Figure 4C and 4D row 3, where pRB1807/811 decreases with BRG1 induction but remains relatively the same when BRG1 induction is blocked. Second, BRG1 is known to be an essential cofactor for RB1/E2F-mediated gene expression [53], and without BRG1, RB1/E2F cannot effectively induce gene expression of target genes required to block G1 to S phase transition. It is not surprising then that we observed that these compounds were not nearly as effective in inducing growth inhibition in these cell lines when BRG1 expression was suppressed as compared with when BRG1 was readily induced. Moreover, we show that the loss of BRG1 expression can cause resistance to TKI-mediated growth inhibition *in vitro* and thus may contribute to the clinically observed resistance to these and other TKIs *in vivo*.

## DISCUSSION

*In vitro* experimental data from a number of different labs have clearly linked both TP53 and RB1 function to the SWI/SNF complex [4, 5]. However, whether TP53 and RB1 are functionally linked to BRG1- or BRM- dependent SWI/SNF complexes has been a subject of debate. Moreover, *in vitro* overexpression experiments are problematic in addressing this question because the strong viral promoters often used yield such high levels that the ectopic expression of BRG1 or BRM activates both BRG1- and BRM-dependent SWI/SNF complexes [15]. Further, shRNA knockdown experiments do not completely deplete BRG1 and BRM in BRG1/BRM- positive cell lines, and low levels of BRG1/BRM can still have an impact on cell phenotypes [23]. It has therefore been difficult to differentiate the separate functions of BRG1 versus BRM. For this reason, the *in vivo* knockout of *Brg1* and *Brm* is better positioned to address the functional differences between Brg1 and Brm. To this end, our data clearly show that Brg1 loss impacts the Rb pathway directly or indirectly and allows tumor cells that harbor hypophosphorylated Rb1 to proliferate as fast as or faster than tumor cells harboring hyperphosphorylated Rb1. RB1 and BRG1 are further linked, as BRG1 loss downregulates CDKs and G1 cyclins to cause a change in RB1 phosphorylation. This change does not cause redistribution of cells in the cycle cell due to growth arrest, as these tumor cells continue to proliferate. The way in which BRG1 regulates cyclins/CDKs is not known, but data from our lab have shown that BRG1 is linked to GSK3β (published data), which regulates the cellular location of p21 and the stability of CCND1 [52, 54, 55].

The relationship between SWI/SNF and RB1 was first demonstrated by experiments performed by Dunaief and Strober [16, 17]. In these experiments, the re-expression of BRG1 induced growth inhibition, which was dependent on RB1, and to a lesser degree, on the RB1 family members p107 and p130. Subsequently, both BRG1 and BRM were shown to possess LXCXE domains that bind RB1 directly [56]. Several labs have shown that BRG1 re-expression can restore RB1 function in BRG1/BRM-deficient cell lines [12, 13]. We and others have shown that restoring BRM expression is equally effective at the restoration of RB1 function [14, 15]. Hence, *in vitro* data have shown that BRG1 and BRM play similar and potentially overlapping roles in the mediation of RB1 function. However, our data strikingly demonstrate that BRG1 loss, but not BRM loss, appears to directly or indirectly contribute to the conversion of RB1 into its hypophosphorylated state. Therefore, BRG1 loss potentially blocks RB1-mediated growth inhibition *in vivo*.

Why is establishing that BRG1 is linked to RB1 important? BRG1 loss is not yet recognized as a major mechanism of RB1 pathway inactivation, in part because BRG1 loss occurs concomitantly with tumor cell loss of CDKN2A (p16) or RB1. Hence, the interdependence of BRG1 on RB1 might be viewed as unimportant to cancer. However, there are a number of drugs used clinically, such as TKIs and CDK inhibitors that inhibit growth in part by activating RB1 [50, 57]. In our previous work, we showed that BRG1 could be silenced by aberrant splicing (irreversibly silenced) or translationally blocked (reversibly silenced) [23], as in the Sum159 and DT13 cell lines. Hence, the treatment of these cancer cells, where BRG1 is reversibly silenced, with TKIs (e.g., Sorafenib and PPP) appears to induce BRG1 and inhibit growth. However, when BRG1 induction is blocked by anti-*BRG1* shRNA knockdown, the cells demonstrate resistance to growth inhibition, which occurs when BRG1 can be induced. It is important to recognize that knockdown by shRNA cannot completely suppress BRG1 expression, and therefore, the loss of growth inhibition may be greater when BRG1 expression is completely abrogated by mutation or aberrant BRG1 splicing. Given this interdependence of BRG1 on RB1, it should not be surprising that BRG1 loss can impart resistance to TKIs (as illustrated by our data). Further research will be needed to determine which TKIs are affected by BRG1 loss.

In contrast to the potential resistance caused by *BRG1* inactivation, a number of different investigators have reported increased sensitivity to taxanes and DNA- damaging agents such as cisplatin and carboplatin [58, 59]. Moreover, as BRG1 is required for double-strand repair, its loss may also enhance the sensitivity of tumors to PARP inhibitors. In support of this assertion is the observation that inactivation of the SWI/SNF subunit ARID1A indeed causes increased sensitivity to PARP inhibitors. Furthermore, BRG1 has been linked to the function of BRCA1, and if true, this could further explain how BRG1 loss might enhance sensitivity to PARP inhibitors. These data help us understand why the connection between BRG1 and RB1 as well as the link between BRG1 and DNA repair have potential clinical consequences in determining sensitivity or resistance to specific drugs used clinically. Hence, BRG1 expression or lack thereof may be an important biomarker to determine which drugs should be clinically used.

Similar to RB1, TP53 functionality has also been linked to SWI/SNF [21], and like RB1, it is unclear if TP53 is functionally linked to BRG1, BRM or both proteins *in vivo*. We showed that *Trp53* mutations occur in our murine model system, which is consistent with other carcinogen-induced murine lung tumor models [60-62]. By Sanger sequencing, we found tumor-driving *Trp53* mutations in both wild type and Brg1-deficient tumors, but *Trp53* mutations were not found in Brm-negative tumors. However, the absence of *Trp53* mutations by sequencing total mRNA does not mean that they were not present, but rather, it indicates that they were below the threshold of detection by Sanger sequencing. Our data demonstrate that *Trp53* mutations are indeed generated by the chemical carcinogen used in this model system in both Brm-positive and Brm-negative tumor cells. When Brm was present, these chemically generated *Trp53* mutations appeared to undergo selection pressure during tumor development, whereas those *Trp53* mutations (typically in the DNA binding domain of Trp53) that lack functionality (Trp53- mediated transcription) were positively selected. This idea is consistent with the oncogene-induced senescence hypothesis, where malignant tumors only arise in those tumor cells where *Trp53* is inactivated. However, we found that, in contrast, malignant tumors arose in Brm- deficient tumors regardless of the type of *Trp53* mutation present in the tumor cells. Our findings indicate that Brm loss appears to usurp the driving force that selects for non-functional Trp53 isoforms. Thus, one can deduce that Brm-dependent complexes are linked to Trp53. As such, one might predict that *Trp53* mutations would not occur concomitantly with *Brm* mutations. However, Brm is not commonly silenced by mutation. It is important, therefore, to remember that Brm is part of a complex, and while Brm itself may not be mutated, other subunits might show a relationship with *Trp53* mutations. This appears to be the case in human ovarian clear cell carcinomas and endometrioid endometrial cancers, where the loss of ARID1A appears to be nearly mutually exclusive from *TP53* mutations [24, 26].

The functional relationship between TP53 and BRM may be important as it may explain how *BRM* polymorphisms are predictive of cancer risk [22], and thus foster early steps of transformation. *BRM* silencing is known to be driven by two insertional 6-base-pair polymorphisms contained within the *BRM* promoter [63]. These polymorphic sites serve as functional binding sites for a number of proteins (HDAC3, HDAC9 and MEF2D) that underlie the epigenetic silencing of *BRM* [64]. As a loss of TP53 function via nonsense or missense mutation is known to be transforming, cancer initiation may occur when *BRM* polymorphisms drive *BRM* silencing, which in turn fosters transformation by thwarting or impairing *TP53* function. In tumors or cell lines, *BRM* silencing is not known to occur concomitantly with *TP53* mutations. The clinical relevance of the link between BRM complexes to TP53 has not been fully delineated. Nevertheless, the connection between BRM complexes and TP53 gives us a deeper understanding of how SWI/SNF is involved in cancer initiation and progression and it generates new questions as to how SWI/SNF is involved in cancer.

## SUPPLEMENTARY MATERIALS FIGURES


