# Supplementary material for: BRG1 and BRM loss selectively impacts RB and P53, respectively: BRG1 and BRM have differential functions in vivo
Source: Oncoscience. 2016 Dec 21;3(11-12):337–50. doi: 10.18632/oncoscience.333 (PMC5235922; doi:10.18632/oncoscience.333)
Supplement: Supplementary file 1 [file oncoscience-03-337-s001.pdf]

SUPPLEMENTARY MATERIALS

Supplementary Figure 1

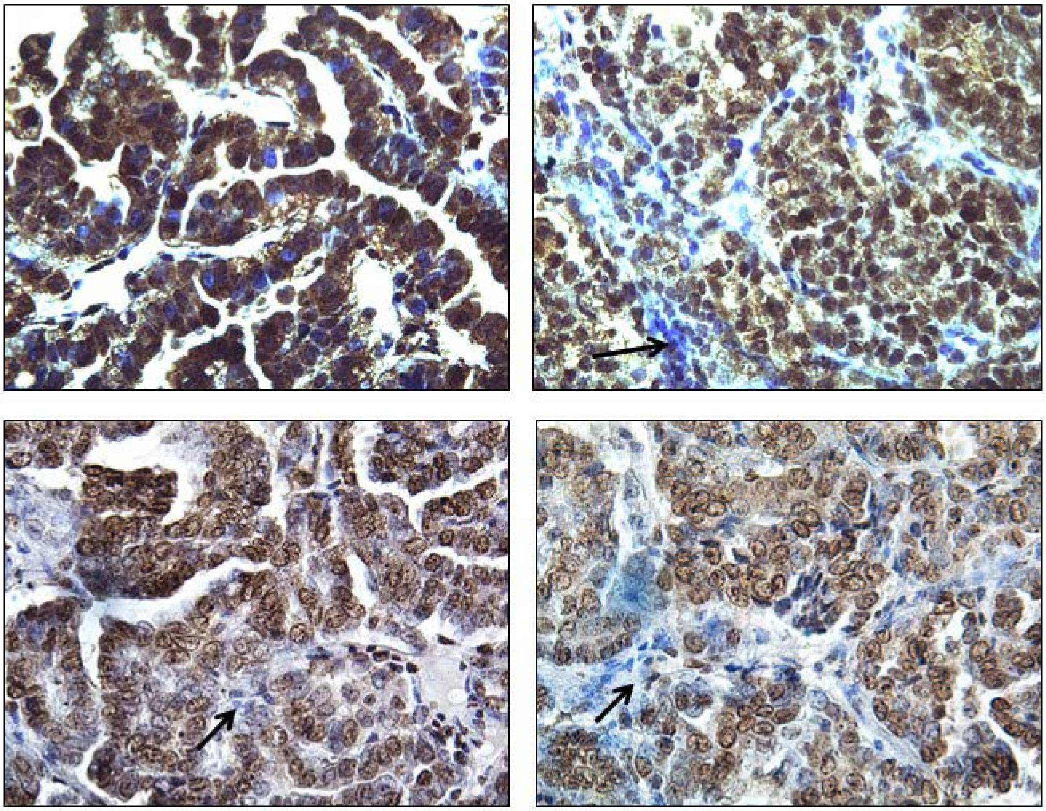

Supplementary Figure 1: p53 Immunohistochemistry. Lung tumors from a WT mouse (a), Brg1KO mouse (b), Brm-null mouse (c), and a DKO mouse (d) all express p53 protein. More than 80% of cells express p53. Microscope bar = 20μM. Arrows indicate interstitial cells that are negative for p53.

Supplementary Figure 2

P53 Mutation	P53 Region	Amino Acid Change
K117E	DBD	Lys117 to Glu
E218G	DBD	Glu218 to Gly
S238P	DBD	Ser238 to Pro
C239R	DBD	Cys239 to Arg
R246STOP	DBD	Arg246 to STOP
F267L	DBD	Phe267 to Leu
E290G	DBD	Glu290 to Gly
M1V	TAD	Met1 to Val
V63A	TAD	Val63 to Ala
T86A	Pro	Thr86 to Ala
W88R	Pro	Trp88 to Arg
R303G	Pro, NLS, C-ter	Arg303 to Gly
E355G	Tet, C-ter	Glu355 to Gly
K383R	C-ter	Lys383 to Arg

Supplementary Figure 2: Seven p53 mutations from the DNA Binding Domain (DBD) and 7 additional p53 mutations located outside the DBD were selected for testing. Each of these mutations were observed to occur in both Brm-positive and Brm-negative tumors. The mutational amino acid change is listed in column 1 (single letter) and column 3 (three letter designation), and the location of each of these mutations with Trp53 is listed in column two.
